# CASE REPORT Successful Treatment of a Rare Massive Dabska Tumor

**Published:** 2013-09-10

**Authors:** Oliver S. Eng, Gregory L. Borah, Christopher J. Gannon

**Affiliations:** ^a^Divisions of General Surgery, Rutgers-Robert Wood Johnson Medical School, New Brunswick; ^b^Plastic Surgery, Rutgers-Robert Wood Johnson Medical School, New Brunswick; ^c^Advanced Surgical Associates of New Jersey, Capital Health Management, Pennington, NJ

**Keywords:** Dabska, tumor, abdominal, reconstruction, resection

## Abstract

**Objective:** The Dabska tumor is a rare low-grade angiosarcoma first described in 1969 by Maria Dabska. Approximately 30 cases of varying presentations have been reported since its initial description. **Methods:** We describe a case of a 23-year-old woman presenting with a massive recurrent left flank hemangioendothelioma, at final resection diagnosed to be an endovascular papillary angioendothelioma (Dabska tumor). The sheer size of the tumor necessitated reconstructive surgery. **Results:** Successful abdominal reconstruction after radical resection of a Dabska tumor was achieved using local fasciocutaneous-type flaps. **Conclusion:** To our knowledge, this is the first case report describing reconstructive surgery following resection of an abdominal Dabska tumor.

The Dabska tumor is a rare low-grade angiosarcoma first described in 1969.^1^ It is characterized microscopically by anastomosing vascular channels with the presence of atypical endothelial cells and papillary luminal projections.^1^^,^^2^ Since its initial description, only approximately 30 cases have been reported. In our report, we describe a patient's massive abdominal Dabska tumor that greatly eclipses, in size, any known reported Dabska tumors of the abdomen (1 cm in diameter in both cases).^3^ The sheer size of the tumor necessitated reconstructive surgery following radical resection.

## PATIENT REPORT

**Figure 1:**
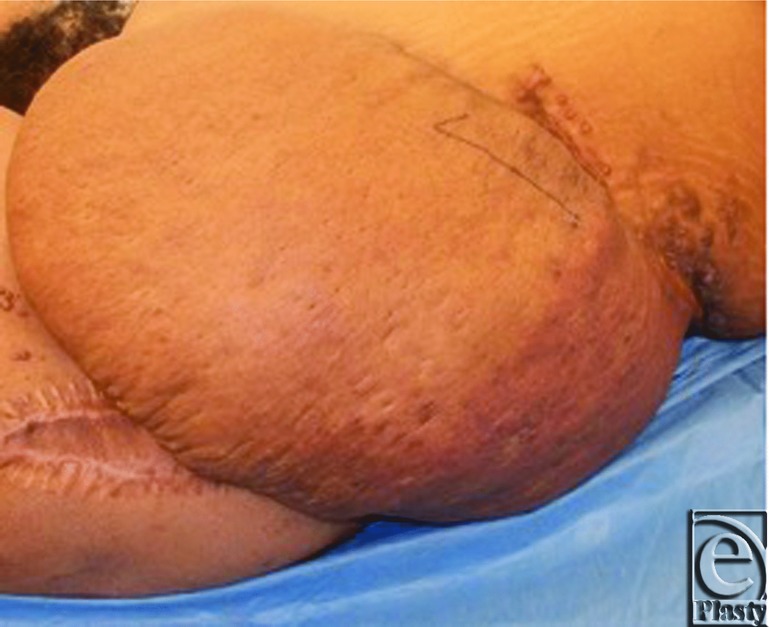
Roughly 40 × 20 cm^2^ left flank Dabska tumor, prior to resection.

A 23-year-old African American woman presented with a roughly 40 × 20 cm^2^ left flank tumor (Fig 1) to the Surgical Oncology Group of Rutgers Cancer Institute of New Jersey. The patient reported having a left-sided hemangioma removed at the age of 3 years but persisted with a port wine stain on her left flank since that time. Approximately 3 years prior to presentation, the patient had her first pregnancy and reported that during her third trimester, she noticed a mass developing on her left flank. During this time, the mass slowly grew to approximately its size at the patient's presentation at our institution. Following delivery of her first child, the patient reported medical evaluation had demonstrated the mass to appear to be a recurrence of her hemangioendothelioma. Following her second pregnancy, the size of her tumor did not significantly change, nor did it affect her pregnancy. It was at this time when she presented to us for evaluation for possible surgical resection after undergoing biopsy showing a Dabska tumor. The patient then presented to the Plastic Surgery Division at Rutgers-Robert Wood Johnson Medical School for evaluation of reconstruction in conjunction with her planned tumor resection.

**Figure 2:**
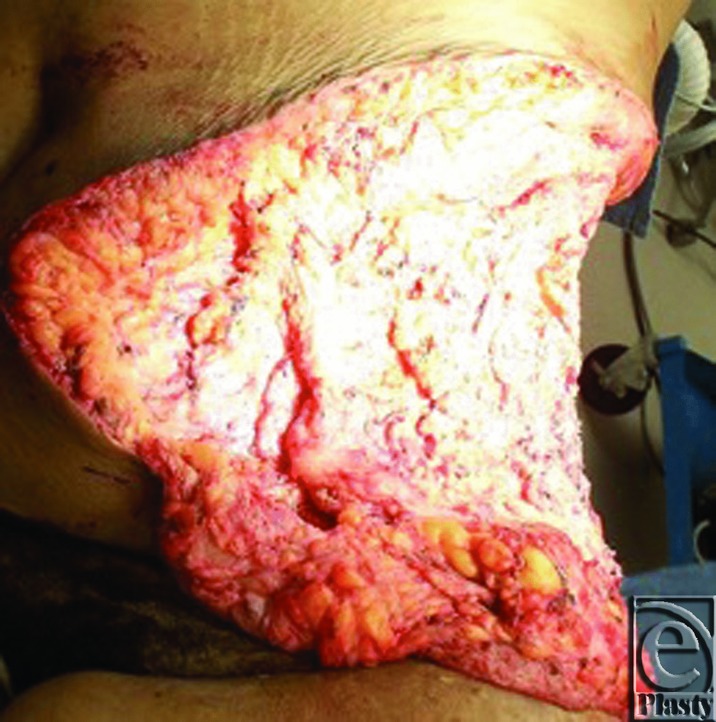
Abdominal defect after radical resection.

**Figure 3:**
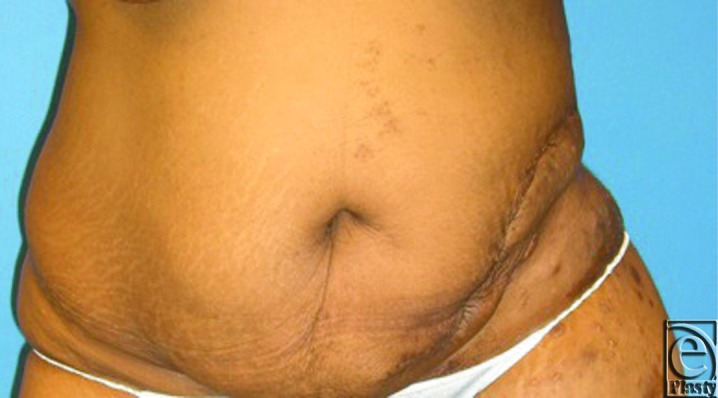
Three-month follow-up, frontal view.

**Figure 4:**
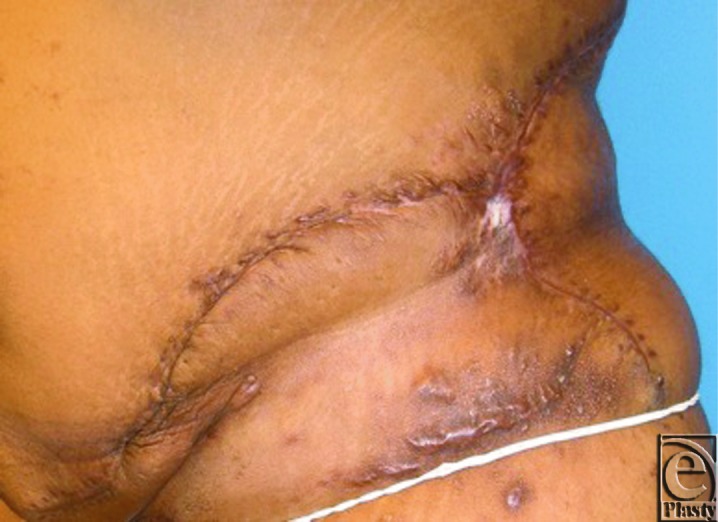
Three-month follow-up, lateral view.

During radical resection, the tumor was dissected down to the abdominal wall fascia, as the tumor did not appear to violate the fascial space. Specimens were submitted intraoperatively to pathology for frozen section, with final diagnosis ultimately showing a 30 × 22 × 4 cm^3^ Dabska tumor. After tumor extirpation, the patient had a defect extending from the umbilicus to the mid-posterior back from the tenth rib to below the hip (Fig 2). Abdominal wall reconstruction was performed with 2 fasciocutaneous-type flaps raised to the level of the costal margin, medially to the umbilicus and posteriorly to the area of the trapezius. Inferiorly, the flaps were extended onto the lateral tensor fascia lata area, posterior to the superior buttock region, and medially into the groin crease. Flaps were advanced and redundancy excised. Flaps were coapted with interrupted 0 and 2-0 polydioxanone sutures for deep and deep dermal components, and multiple subcutaneous drains were placed. Epidermal coaptation was achieved with multiple stainless steel staples. The patient tolerated the procedure well and healing was uneventful. After discharge, the patient was largely noncompliant but returned for drain management and staple removal in the initial postoperative visit. She returned for one late follow-up appointment 3 months postoperatively with excellent reconstructive results (Figs 3–4). The patient has not returned for later follow-up since the 3-month postoperative visit despite vigorous outreach attempts.

## DISCUSSION

It has been shown that early involvement of a plastic surgeon in the surgical intervention of soft tissue sarcomas increases the likelihood of functional restoration and satisfactory wound healing.^4^ This is the first reported case of reconstructive surgery following resection or excision of an abdominal Dabska tumor. Our comprehensive literature review yielded only 1 case report on reconstructive surgery following surgical excision of a recurrent pedal retiform hemangioendothelioma.^5^ This occurred in an 8-year old female patient with a 2.3 cm mass within the dorsal subcutaneous soft tissues of the right foot between the first and second metatarsophalangeal joints (MTPJs). Of note, the patient had undergone a prior excisional biopsy 3 years prior, demonstrating hobnail (Dabska retiform) hemangioendothelioma. The patient underwent repeat excisional biopsy, yielding the same diagnosis. The patient then returned for follow-up every 3 months for physical examination and magnetic resonance imaging; at 12 months, a 2.9-cm lesion between the first and second MTPJs was detected on magnetic resonance imaging, suggestive of recurrence. In an effort to preserve her forefoot, the patient underwent resection of the entire mass with a margin of surrounding subcutaneous tissue, followed by a fillet of second digit flap.^5^ She recovered well from the surgery and had not displayed evidence of recurrence at the time of publication of that report.^5^

With only approximately 30 reported cases of the Dabska tumor in the literature, 60% of patients were in children, with no predilection for either gender.^1^^,^^6^ Ages of patients at presentation range from birth to 83 years.^3^^,^^7^ Various anatomic locations of lesions have been described, most often presenting in the oral cavity and bones, and some of which include extremities, abdomen, spleen, and testis.^2^^,^^8^^-^10 Most skin and palpable tumors have come to medical attention when they are 2 to 3 cm in diameter, with 1 buttock tumor reported to grow to 40 cm^3^. The treatment of choice is wide local excision (with regional lymphadenectomy when those structures appear involved).^11^^,^^12^

Dabska tumors have a generally favorable prognosis, as most patients have not demonstrated evidence of recurrence with follow-up periods ranging from 4 to 30 years.^3^ Upon a 30-year review of Dabska tumors and the outcomes of the original 6 patients reported (all of whom had undergone wide local excision), 5 patients did not experience recurrence, while 1 patient died of pulmonary metastases.^3^ Protocols for not only surgical removal but also surveillance and follow-up have yet to be established, but it is reasonable to presume that obtaining a chest radiograph for any Dabska patient with pulmonary symptoms should be considered.

Our patient demonstrates a successful abdominal reconstruction after wide local excision of a massive abdominal Dabska tumor using local fasciocutaneous-type flaps. Our patient was obese and had substantial areas of trunk and thigh skin that were somewhat auto-expanded by the gravitational droop of the tumor. We were able to mobilize the abdominal wall tissues at the level of the external oblique fascia and rectus prefascia plane extensively to provide wound coverage over the large defect area of the resection. An attempt was made to utilize the superior flap tissue as much as possible to preserve optimum vascular inflow to the large flaps. Our presumption was that the massive vascular nature of the lesion might have improved flap blood flows compared to normal skin. This vast area of undermined skin and fascia required extensive subcutaneous drainage by multiple drains to prevent seroma formation, which could have complicated the postoperative recovery period. Drains were maintained until output was less than 30 mL per day per drain. We were fortunate that intramuscular extension by the tumor was not seen, and this pattern appears to be rare from our review of the literature. Much remains to be elucidated about the pathophysiology of the Dabska tumors, and we look forward to future reports to aid our understanding of these rare tumors.
